# Flexoelectric effect in an in-plane switching (IPS) liquid crystal cell for low-power consumption display devices

**DOI:** 10.1038/srep35254

**Published:** 2016-10-12

**Authors:** Min Su Kim, Philip J. Bos, Dong-Woo Kim, Deng-Ke Yang, Joong Hee Lee, Seung Hee Lee

**Affiliations:** 1 Liquid Crystal Institute, Kent State University, Kent, OH 44242, United States; 2 Applied Materials Institute for BIN Convergence, Department of BIN Convergence Technology, and Department of Polymer Nano Science and Technology, Chonbuk National University, Jeonju, Jeonbuk 561-756, Korea

## Abstract

Technology of displaying static images in portable displays, advertising panels and price tags pursues significant reduction in power consumption and in product cost. Driving at a low-frequency electric field in fringe-field switching (FFS) mode can be one of the efficient ways to save powers of the recent portable devices, but a serious drop of image-quality, so-called image-flickering, has been found in terms of the coupling of elastic deformation to not only quadratic dielectric effect but linear flexoelectric effect. Despite of the urgent requirement of solving the issue, understanding of such a phenomenon is yet vague. Here, we thoroughly analyze and firstly report the flexoelectric effect in in-plane switching (IPS) liquid crystal cell. The effect takes place on the area above electrodes due to splay and bend deformations of nematic liquid crystal along oblique electric fields, so that the obvious spatial shift of the optical transmittance is experimentally observed and is clearly demonstrated based on the relation between direction of flexoelectric polarization and electric field polarity. In addition, we report that the IPS mode has inherent characteristics to solve the image-flickering issue in the low-power consumption display in terms of the physical property of liquid crystal material and the electrode structure.

The name flexoelectricity points out a connection between a geometric deformation of the director field and the electric polarization on liquid crystals. Understanding of this phenomenon is crucial to explain some effects, which are not as usual as the dielectric effect^1^. Considering the symmetry arguments, nematic liquid crystals are commonly described as a rod-like body with indistinguishable head and tail; however, the asymmetric shape and the electrical polarity in single molecule become important where the non-polar nematic symmetry is broken, i.e., if the head of a molecule is arranged in one direction. When combination of broken nematic symmetry and molecular asymmetry is associated with director (average orientation) deformation, the net polarization arises,


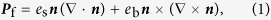


where ***n**, e*_s_ and *e*_b_ denote unit vector of liquid crystal director, splay and bend flexoelectric coefficients, respectively^2^. The meaning of the formula is that the flexoelectric polarization appears by linear coupling between splay or bend deformation and the values of *e*_s_ or *e*_b_, which correspond to both the shape and the electrical polarity of molecules. The statistical approach based on the maximum molecular packing condition gives rise to the *e*_s_ = *α*(2*μ*_||_*K*_11_/*k*_*B*_*T*)(*N* · *a*/*b*)^1/3^ and *e*_b_ = *α*(*μ*_⊥_*K*_33_/2*k*_*B*_*T*)(*N* · *b*/*a*)^2/3^, where *α*, and *a*/*b* are angle and aspect ratio of molecular anisotropy; *μ, k*_B_, *T*, and *N* are dipole moment, Boltzmann constant, temperature, and molecular excess number (the electric polarization *P* = *Nμ*)^3^,^4^,^5^. In experiment, on the other hand, the quantitative measurement of the flexoelectricity requires both robust and sensitive experimental sets; there has been many interesting works measuring the quantities^6^,^7^,^8^. Reports on the flexoelectric coefficient with bent-core molecules interestingly exhibits the order of *e*_b_ ~ 50 nC/m^7^,^9^, while it is relatively small based on the calamitic liquid crystal molecule: the order of 10 pC/m^3^,^4^,^6^,^8^,^10^,^11^. Such measurements are executed by generating the director deformation by both mechanical strain and biased external electric fields.

After Meyer discovered the flexoelectric effect^1^, Prost and Pershan executed an experiment to observe it by applying voltage via interdigitated electrodes to a homeotropically aligned cell^12^. The result shows that the dielectric and flexoelectric effects of calamitic liquid crystals are optically distinguishable under the context of the lateral electric field and vertical optic path. Such a system consists of three energetic terms: elastic free energy density *f*_elas_, quadratic dielectric coupling *f*_dielec_, and linear flexoelectric coupling *f*_flexo_.

In order to reduce the free energy of system, the elastic deformation occurs to compensate the cost of dielectric and flexoelectric effects. Unlike the mechanical strain induced director deformation, the contribution of the external electric field to break the orientational symmetry depends on driving frequency when applying the alternating current field. If the frame duration of driving frequency is faster than the physical response of the liquid crystal molecules, no orientational symmetry would be broken and only dielectric response (quadratic coupling) takes place. However, even at lower frequencies, where the flexoelectric coefficients are non-zero, the viscosity-limited director field response can cause the flexoelectric effect to be unobservable.

In liquid crystal displays, usually the driving frequency is 60 Hz or higher; it seems no flexoelectric effect has been an issue from such a device application perspective. Recently, however, the scientific interests^1^,^11^,^13^,^14^,^15^,^16^ in the flexoelectricity induced by the external electric field^4^,^10^,^14^ are exposed to the practical display applications and actively explored under low-frequency driving (below 30 Hz) with fringe-field switching (FFS) mode for saving power consumption^17^,^18^,^19^,^20^,^21^,^22^. As above mentioned, the flexoelectricity of the calamitic liquid crystal has been known as small effect; however, the optical property associated with such small effect turns out to be remarkably significant even an image-flickering being noticed by human eyes because the voltage-dependent transmittance curves, as well as the time-dependent transmittance curve, of positive and negative field frames in the FFS mode are deviated each other due to collective splay and bend deformation^8^,^13^,^23^. Furthermore, despite of the reasonable studies according to the detailed mechanism of the flexoelectric effect in the FFS mode, inconsistency of the local transmittance curves among the reported results has been found, especially in the region above each electrode^18^,^19^,^20^,^21^. In the FFS mode, a stack of one transparent electrode and another transparent interdigitated electrode is separated by an insulation layer; however, in in-plane switching (IPS) mode, only one layer of a transparent electrode is interdigitated so that the electric field shape is much simpler than that in the FFS mode. This simple field formation is relatively intuitive to analyze upon the existence of the flexoelectric effects.

At this point, we refresh our attention to the IPS mode whose flexoelectric effect has not been reported from the perspectives of not only the image-quality but also the detailed electro-optical analysis. We expect the IPS mode can be used in various large-size panels for static images of the low-power consumption besides the FFS mode. Although the FFS mode is widely used in display panels of portable devices^24^,^25^, its large self-forming storage capacitor obstructs the application to large-size panels. As well-known, the main elastic deformation spotlighted in the IPS mode is the twist deformation between electrodes by an in-plane electric field; however, there is no local collective splay and bend deformations so that this twist-dominant driven mechanism might have hindered to expect if the flexoelectric effect would be significant in the IPS mode.

At present, the IPS liquid crystal display is a common light modulation method in TV and desktop monitors because it exhibits wide-viewing angle property owing to the in-plane rotation although it has inherently low-transmittance and high-driving voltage^26^. More recently, these shortcomings can be overcome by narrowing down the electrode width (space) *w (l*) because the driving voltage is linearly proportional to *l* in the IPS mode. Where *l* ~ *d*, the lateral-field-dominant system becomes the lateral and vertical fields dominant system so that the elastic deformation near and above electrodes will be of more splay state than that where *l* > *d*. Therefore, one might expect non-negligible flexoelectric effect in the IPS mode. Where *l* < *d*, the director can rotate not only between electrodes but also even above electrodes (which of the area has no lateral field to contribute to twist deformation at all); therefore, it gives rise to very high-transmittance even comparable to that in the FFS mode. This is because, in dynamic time-evolution upon the response of the electric field, the elastic torque is transferred to neighboring liquid crystal molecules, hence the twist deformation can occur on the center of electrodes. Thus, the flexoelectric effect in the IPS mode is expected to become more important and it should be thoroughly investigated.

However, where the flexoelectric effect takes place with such electrode condition *l* < *d*, the splay (bend) deformation on the edge (center) of electrodes seems to result in counter-intuitive behavior as well as the local transmittance curves upon the nematic head-tail symmetry breaking at low-frequency driving and the balance among the free energy of the elasticity, dielectricity, and flexoelectricity. Therefore, the flexoelectric effect in such a context should be deeply and quite urgently explored and understood.

## Results

**Figure 1:**
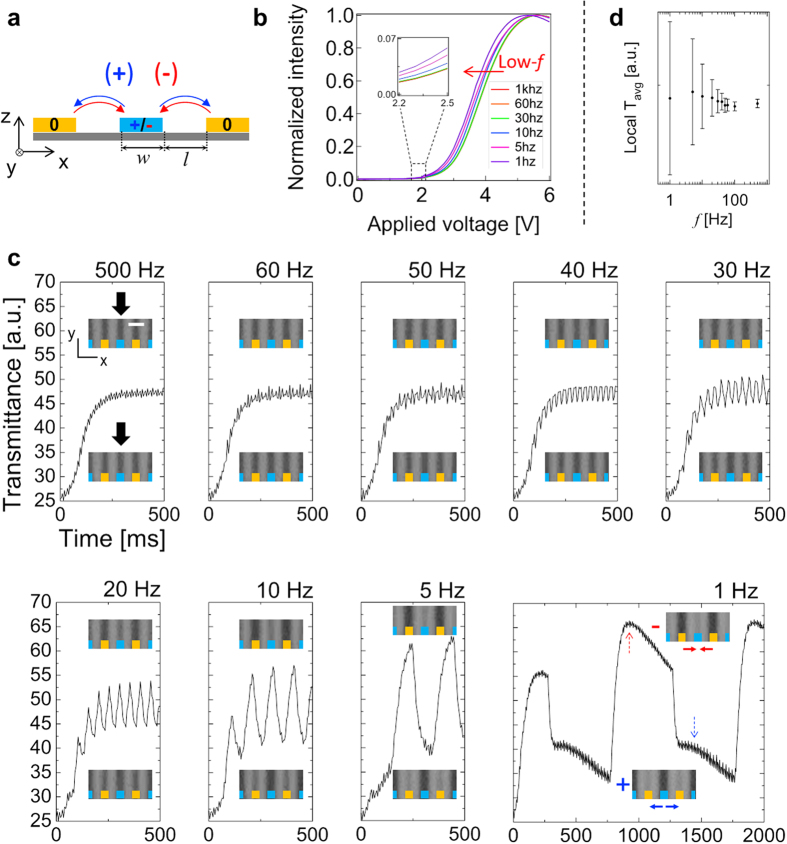
Experimental observation of flexoelectric effect in in-plane switching (IPS) liquid crystal cell. (**a**) Schematics of the cell geometry (Blue and red arrows represent electric field vector). (**b**) Voltage-dependent transmittance curves with respect to various driving-frequencies. (**c**) Local time-dependent transmittance on the indicated location by black arrows under the application of ~3.7 V (50% of the maximum transmittance). Insets: POM images with scale bar, 10 μm. (**d**) The local time-averaged transmittance after the curves saturated and its standard deviation with respect to the driving frequency *f*. (See Supplementary Videos S1 and S2 for observation of local transmittance shift at *f* = 60 Hz and 10 Hz, respectively).

We prepare a unit cell with the IPS mode as schematically illustrated in Fig. 1(a) which the blue and red arrows indicate the direction regarding the polarity of the applied electric fields. As the curves are plotted in Fig. 1(b), the driving-frequency *f* gets lowered, the threshold and operating voltages reduced. In order to verify the reason of the reduction, local time-dependent transmittance at the location indicated by the black arrow are measured, and polarizing optical microscopy (POM) images are observed as shown in Fig. 1(c). The head-tail symmetry of nematic liquid crystal becomes broken as the reciprocal of the driving frequency is longer enough to the liquid crystal molecular response (head-tail flipping); thus, the periodicity of the local transmittance becomes spatially shifted and apparently noticed. As clearly shown in the inset of Fig. 1(c) at 1 Hz, the local transmittance is shifted toward along the electric field vectors indicated by red and blue arrows. The fluctuation of the local transmittance is more obviously demonstrated by both average and standard deviation with respect to the driving-frequency *f* as shown in Fig. 1(d). The larger standard deviation implies much significant transmittance fluctuation over the time. Note that below *f* ≈ 50 Hz (1/*f* ≈ 20 ms) the liquid crystal molecules have sufficient interval to physically flip over its head-to-tail. The frequency-dependence of the spatial fluctuation of the local transmittance is obviously shown in Supplementary Videos S1 and S2 at *f* = 60 and 10 Hz, respectively, and remarkably, although it is small, the fluctuation exists even at 60 Hz.

**Figure 2:**
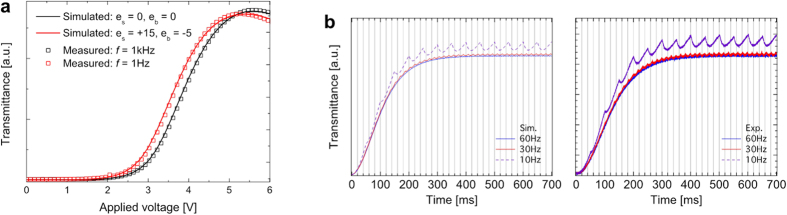
Measured and numerically simulated static and dynamic responses. (**a**) Voltage-dependent transmittance with/without flexoelectricity, and (**b**) time-dependent transmittance with the applied voltage at 50% of the maximum transmittance with respect to various frequencies: 60, 30, and 10 Hz.

Numerical simulation can provide details in the optical effect regarding the director field and flexoelectricity. In order to verify the appearing phenomenon, thorough observation of consistency between the experiment and numerical simulation was done. In this study, rather than vary the frequency of the applied field, the effect of this parameter was simulated by changing the value of the flexoelectric coefficients. Figure 2 shows the measured and simulated both the static and dynamic responses: (a) voltage-dependent transmittances and (b) time-dependent transmittance. In Fig. 2(a), the transmittances measured at 1 kHz and 1 Hz are in excellent agreement with the simulated transmittance with flexoelectric coefficients *e*_s_ (*e*_b_) = 0 (0) pC/m and 15 (−5) pC/m, respectively; the mechanical, electrical, and optical parameters of the liquid crystal used in the simulation are identical with that used in the experiment. With the applied voltage for 50% of the maximum transmittance as shown in Fig. 2(b), the transmittance fluctuation becomes significant as the driving-frequency gets lowered and the average transmittance is higher at lower-frequency than at higher-frequency, which is also in a good agreement between numerical and experimental results.

**Figure 3:**
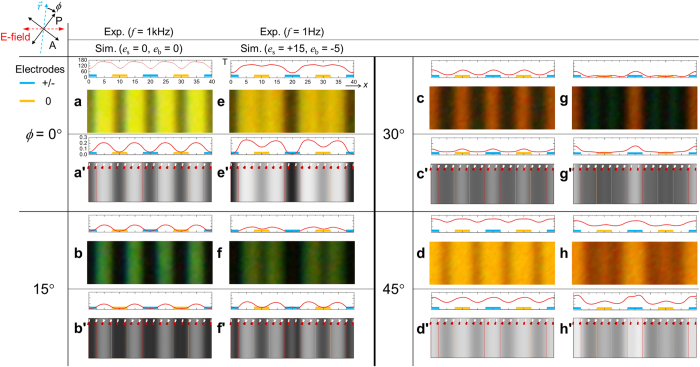
Optical appearance of flexoelectric effect comparing between experiment and numerical simulation. (**a–d**,**a′–d′**) Without and (**e–h**,**e′–h′**) with flexoelectricity. (**a–h**) Polarizing optical microscopy (POM) images and (**a′–h′**) numerically simulated transmittance images (gray scale) at various angles *ϕ* between rubbing direction and one of the transmitting axes of crossed polarizers.

In a previous study^23^, deformation of liquid crystal director gives rise to spatial oscillation of refractive index; the pitch of an optical diffraction grating, which emerge by the quadratic and linear couplings, correspond to half of and equal to the repeated displacement (*w* + *l*), respectively. Accordingly, the pitch of the repeated director deformation is equal to and twice of (*w* + *l*), respectively. Similarly, in Fig. 3, the space-resolved transmittance up on the dielectric and the combination with flexoelectric effect are observed and simulated. The same experiment and simulation sets as in Fig. 2 are represented with POM images and calculated transmittance with the same wavelength spectrum characteristic of a tungsten-halogen lamp in POM. Each row shows the data sets up on the rotation of the optic axis of liquid crystal to the crossed polarizers by the angles *ϕ* = 0°, 15°, 30°, and 45°. The overall images and space-resolved transmittance curves show in good agreement although small details are deviated as in Fig. 3(f,f′,h,h′). As clearly shown in the images in Fig. 3, the appearance of the local transmittance is symmetric with respect to the middle between the electrodes without flexoelectricity, but is asymmetric with flexoelectric effect, that is, the repeated optical pitches are equal to and twice of (*w* + *l*), respectively.

**Figure 4:**
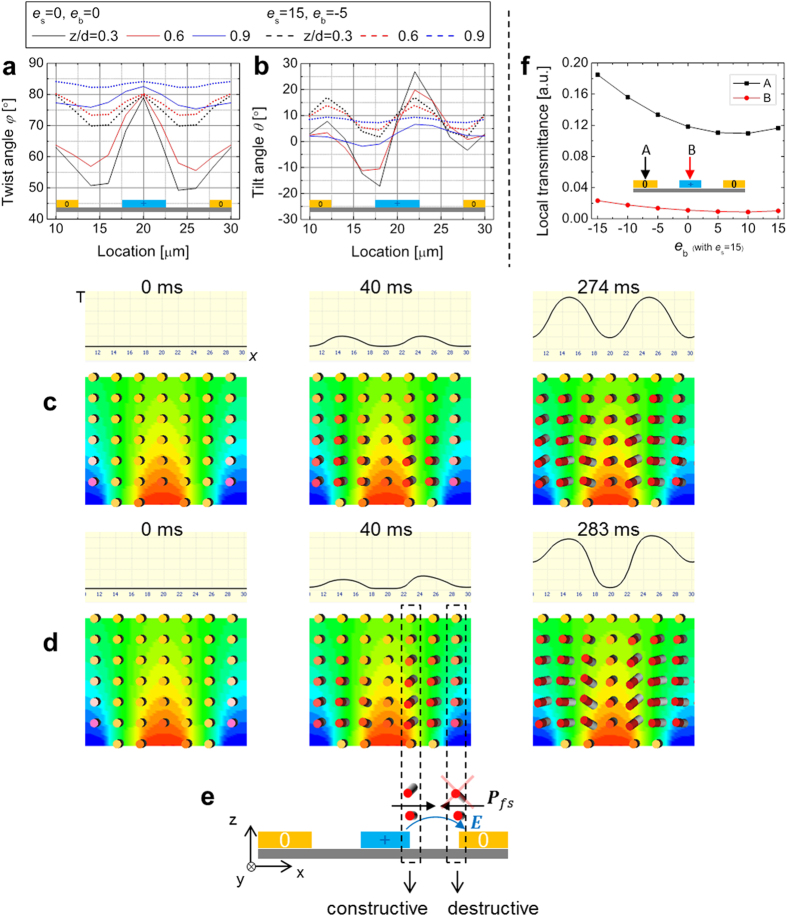
Analysis on the director field and local transmittance under flexoelectric effect. Numerical simulation of (**a,b**) angles of twist and tilt deformations with respect to the location (see Supplementary Figure S2), time- and space-resolved transmittance and director fields (**c**) without and (**d**) with flexoelectricity (see Supplementary Videos S3 and S4 for (**c,d**), respectively). (**e**) Schematic interpretation on director field formation along the coupling between the applied electric field and the flexoelectric effect. The direction of flexoelectric polarization from splay deformation and the applied electric field are constructively or destructively associated on the edge of electrodes; the red-cross indicates no splay. (**f**) The local transmittance depending on variation of *e*_b_ with fixed *e*_s_ = 15 pC/m.

According to the dipole moment along parallel or perpendicular to the long axis of a molecule, one can expect the local transmittance with flexoelectric effect would depend on the flexoelectric coefficients due to the electric field formation associated with the director distortion. Figure 4 shows the numerical simulation result at 50% of the maximum transmittance, which the local transmittance and director field at the maximum transmittance is also shown in Supplementary Figure S1. As shown in Fig. 4(a), the value of *e*_s_ and *e*_b_ contributes to the twist and tilt angles, hence influencing the local transmittance. The schematics of the electrodes indicate + and 0 potential with blue and orange colors in Fig. 4. When the *e*_s_ (*e*_b_) = 0 (0) pC/m, similar degree of twist deformations occurs above the + and 0 electrodes; however, up on the *e*_s_ (*e*_b_) = 15 (−5) pC/m, the twist deformation more significantly takes place above the 0 electrode than the + electrode as shown in Fig. 4(a). Likewise, in Fig. 4(b), but in opposite sense, the tilt deformation is more significant above the + electrode than the 0 electrode (see Supplementary Figure S2 for more information). The calculated director fields are visualized with time-resolved local transmittance in Fig. 4(c,d). As schematically described in Fig. 4(e), the flexoelectric polarization regarding splay ***P***_*fs*_ is in same direction (constructive) with respect to ***E*** above the edge of the + electrode, but in opposite direction (destructive) above the edge of the 0 electrode.

From the free energetic perspective, the *f*_elas_ is in opposite sign with *f*_flexo_. When constructive, the flexoelectric effect (in the restricted location described in Fig. 4(e): the splay is dominant) can be pursued by the elastic distortion compensating each other aiming to reduce the free energy. However, when destructive, the *f*_flexo_ becomes the same sign as the *f*_elas_ because the direction of vectors become opposite between ***P***_*fs*_ and ***E***, so that the flexoelectric effect is suppressed. In this circumstance, the twist deformation becomes dominant to compensate the existing *f*_dielec_ instead of the *f*_flexo_; the reason of no bend deformation would be that the cost of twist is lower than that of bend deformation. This result can be similarly applied to the coupling between the bend deformation and the electric field direction perpendicularly associated with the long axis of molecules, which mostly takes place above the middle of the electrode location. Figure 4(f) shows the local transmittance above the middle of the electrode locations A and B with variation of the *e*_b_ with fixed *e*_s_ = 15 pC/m. The local transmittance significantly increased on the location A (destructive) when |*e*_b_| becomes large, while it changes small degree on the location B (constructive).

**Figure 5:**
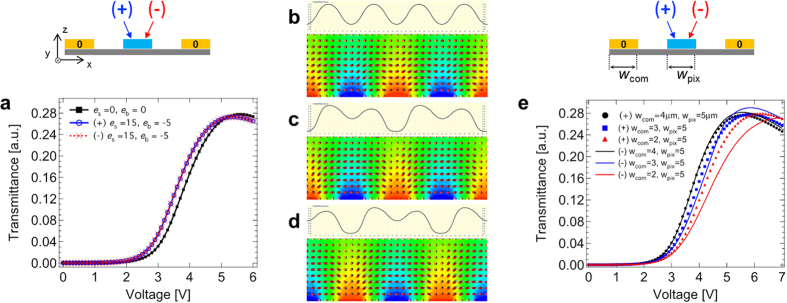
Electrode-structural analysis on the in-plane switching (IPS) mode. Numerical simulation of (**a**) the voltage-dependent transmittances and (**b–d**) the local transmittance and director profiles, where (**b**) *e*_s_ = *e*_b_ = 0 pC/m and (**c**) positive, (**d**) negative frames with *e*_s_ = 15 pC/m, *e*_b_ = −5 pC/m at 3.7 V (50% of the maximum transmittance); *w (l*) = 5 (5) μm. (**e**) The voltage-dependent transmittances where varying common electrode width *w*_com_ with fixed pixel electrode width *w*_pix_ = 5 μm.

The voltage-dependent transmittance in Fig. 5(a), which is the integral of the duration and the region below the curves in Fig. 5(c,d), is the same for both the positive and negative frames although the local transmittance curves are not the same for both frames. This is because, owing to the electrode geometry of the IPS mode, the shape of the electric fields in the positive frame is identical to the lateral inversion of that shape in the negative frame as illustrated in Fig. 1(a). In order to verify it, the voltage-dependent transmittances in the positive and negative frames are simulated while the width of only common electrode varies (*w*_com_ = 4, 3, and 2 μm) as shown in Fig. 5(e). As the (*w*_pix_ − *w*_com_) gets bigger, the deviation of the voltage-dependent transmittance curves becomes significant. Upon the general spatial resolving power of human eyes, the local transmittance difference is not distinguished by the human eyes’ view, so that theoretically, the overall transmittance is not fluctuating as long as the voltage-dependent transmittance is the same, hence no image-flickering takes place with the IPS mode. Here, a more common criterion for the judgement of the image-flickering can be proposed, that is, focus on how significantly the behavior of the local transmittance curves is deviated between positive and negative frames regardless of the location. Therefore, the IPS electrode structure can be advantageous for regarding issues.

**Figure 6:**
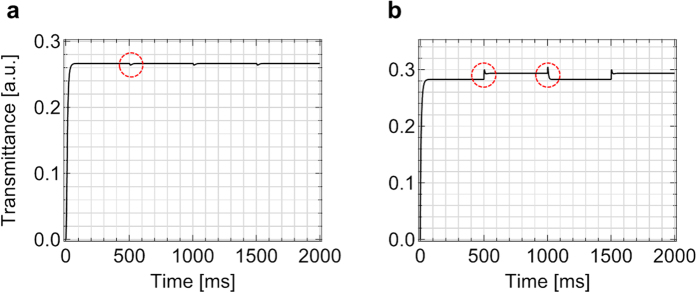
Verification of the dynamic transmittance fluctuation upon altered field-polarity. Numerical simulation of the time-dependent transmittances of (**a**) in-plane switching (IPS) and (**b**) fringe-field switching (FFS) modes with relatively high-dielectric anisotropy and low-viscosity liquid crystal (than the liquid crystal used before this result) when applying voltage for 100% of transmittance. The red-dotted circles indicate the transmittance fluctuation during the transient moment of the electric field polarity. The flexoelectric coefficients are *e*_s_ = 15 pC/m, *e*_b_ = −5 pC/m for both (**a**,**b**); *w (l*) = 5 (5) μm in (**a**) and 3 (4) μm in (**b**).

However, besides the static electro-optic characteristic, the dynamic electro-optic behavior may cause transmittance fluctuation owing to the altered field-polarity and it can also damage the quality of images. This can be verified by time-dependent transmittance curves as shown in Fig. 6. In this numerical simulation, we introduce relatively high-dielectric anisotropy (Δ*ε* ~ 15) and low-rotational viscosity (*γ*_1_ ~ 90 mPas) liquid crystal material, which is commercially available and used in a liquid crystal display panel of television sets. The behavior of the time-dependent transmittance curve in the IPS mode shows almost the same saturated transmittance between the altered frames as shown in Fig. 6(a) with the frame-transient duration less than 50 ms and the slight transmittance shaking by only ~1.2% point. This result clearly demonstrates that the degree of the image-flickering as well as the transmittance fluctuation is quite low in the IPS mode.

In addition, the structural advantage of the IPS mode upon the flexoelectric effect can be verified by comparing with the FFS mode (the electrode structure is a stack of patterned pixel electrode with *w (l*) = 3 (4) μm, a plane common electrode, and an insulation layer, which separates the pixel and common electrodes) with the same material condition as shown in Fig. 6(b). Although both the IPS and FFS modes show similar duration of the transmittance fluctuation, the saturated transmittance for positive and negative frames in FFS mode is different by ~3.7% point, which means, transmittance difference of ~3.7% will be continued for the most of duration in each frame and this will be altered by every frame. Furthermore, the transmittance fluctuation at the moment of the field-polarity change is ~5.8% and ~7.8% for positive and negative frames, respectively. This result shows the electrode structure of IPS mode shows excellent advantages for image-quality under the flexoelectric effect.

**Figure 7:**
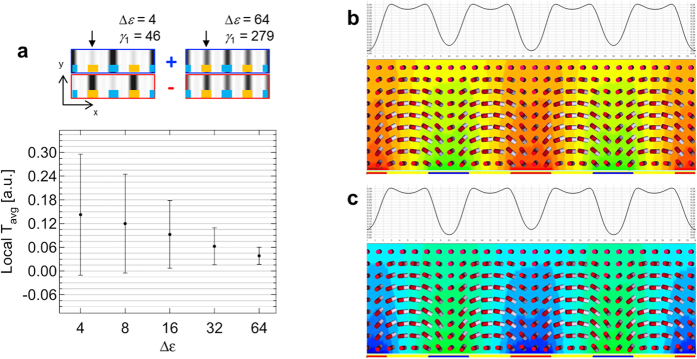
Suppression of flexoelectric effect by changing the dielectric anisotropy of liquid crystal. Numerical simulation of (**a**) the transmittance images with Δ*ε (γ*_1_) = 4 (~46 mPas) and Δ*ε (γ*_1_) = 64 (~279 mPas) at positive and negative frames, and the averaged local transmittance and its standard deviation with respect to Δ*ε* (black arrows indicate the location of the fluctuating local transmittance). (**b,c**) The local transmittance and director profile with Δ*ε (γ*_1_) = 64 (~279 mPas) at (**b**) positive and (**c**) negative frames at the applied voltage for 100% of the maximum transmittance in voltage-dependent transmittance curve.

Considering the free energetic description of the system with the Eq. (2, 3, 4) as above analyzed in Fig. 4, the local director deformation is somehow counter-intuitively influenced by the applied electric field because of such quadratic and linear contributions of the effects, the association of the flexoelectric polarization and electric field directions. Thus, we vary the Δ*ε* and *γ*_1_ of the liquid crystal by following the linear correlation between Δ*ε* and *γ*_1_^27^ as the results are shown in Fig. 7. Remarkably, as the Δ*ε* and *γ*_1_ become high, the local transmittance fluctuation on the region above electrodes becomes significantly reduced, and also, the behavior of the local transmittance curves for both positive and negative frames gets similar as well as the director deformation.

Taking a look back to the graph in Fig. 1(d), the similar tendency is shown between the experiment and simulation in Fig. 7(a). The fluctuation with Δ*ε (γ*_1_) = 4 (~46 mPas) dramatically drops by approximately 86% point with Δ*ε (γ*_1_) = 64 (~279 mPas), and the local transmittance images above the plot in Fig. 7(a) clearly show that the degree of brightness change by the location is significantly reduced as Δ*ε* and *γ*_1_ increases. This result is also confirmed in Fig. 7(b,c) with local transmittance curves with respect to the location. The director profile shows that the destruction on the splay and bend deformations, in terms of the opposite direction between the flexoelectric polarization and the electric field, is restrained. Therefore, the splay and bend deformations tend to pursue regardless of the flexoelectric effect at low-frequency driving. As a result, we confirmed that even the local transmittance difference, that is, the flexoelectric effect, can be suppressed by increasing Δ*ε* and *γ*_1_ of liquid crystals.

**Figure 8:**
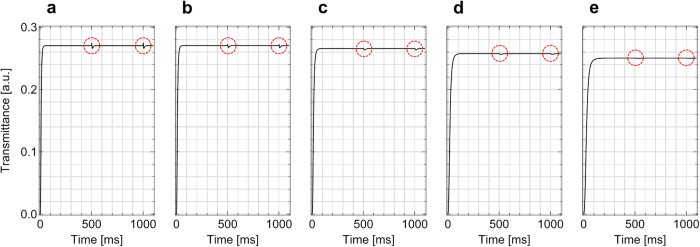
Reduction of transmittance fluctuation under flexoelectric effect by changing the dielectric anisotropy. Numerical simulation of the time-dependent transmittance with (**a**) Δ*ε (γ*_1_) = 4 (~46 mPas), (**b**) 8 (~62 mPas), (**c**) 16 (~93 mPas), (**d**) 32 (~155 mPas), and (**e**) 64 (~279 mPas) at *f* = 1 Hz with the applied voltage at 100% of the maximum transmittance. The red-dotted circles indicate the degree of transmittance shaking during the transient moment of the electric field polarity.

Finally, the transmittance fluctuation is also evaluated by the time-dependent transmittance as a function of both Δ*ε* and *γ*_1_ as shown in Fig. 8. The result in Fig. 8(c) used similar values of Δ*ε* and *γ*_1_ in Fig. 6. Remarkably, the transmittance shaking during the polarity transition, which is indicated by the red-dotted circles, is significantly reduced as Δ*ε* and *γ*_1_ increase, although a certain degree of the saturated transmittance decreases and the response time increases partially as shown in Fig. 8(e). The result shows significant reduction on both the image-flickering and the transmittance fluctuation in dynamics at the low-frequency driving.

## Discussion

We have thoroughly analyzed the effect of flexoelectricity in nematic liquid crystals when lateral electric fields are applied in IPS mode. In this system configuration, the coupling among the director distortion, dielectric effect, and flexoelectric effect differently occurs on the location, corresponding to the direction of the electric field vector. As a result, the local transmittance is influenced by constructive or destructive association between the directions of the electric field and flexoelectric polarization. In the constructive association, flexoelectric effect is pursued as well as the splay and/or bend director deformation whereas the flexoelectric effect is suppressed in the destructive association. In the destructive association, the twist deformation dominantly takes place than the splay or bend deformation.

One may state that the local transmittance curve dropping in Fig. 1(c), clearly noticed at 1 Hz, would be caused by charge impurities in the cell^8^. It seems obvious that the charge impurity and flexoelectricity cause different behaviors of the time-dependent transmittance curves. For instance, the spatial fluctuation of the local transmittance corresponds to the flexoelectric effect whereas the transmittance dropping up on one applied electric polarity reveals charge impurity effect as shown in Supplementary Figure S3. Here the crucial importance of this result arises because the analysis of the time-dependent transmittance becomes complicated owing to mixed experimental information from the flexoelectric effect and the charge impurity effect. Moreover, this transmittance dropping will significantly contribute to the image-flickering regardless of the flexoelectric effect, as in common for most of liquid crystal displays. Therefore, this issue should be carefully addressed in practical applications.

As shown in Supplementary Video S1, the local transmittance shift is observed due to the flexoelectric effect even at *f* = 60 Hz. This finding not only means that the flexoelectric effect in liquid crystals continuously exists regarding *f*, but also supports our demonstration that the image-flickering in the IPS mode would not be significant regardless of the driving frequency. Where the optical periodicity of the local transmittance is not distinguishable by the general space- and time-resolving power of human eyes and assuming no ions and impurities in the system, the image-flickering is not noticeable in the IPS mode.

The theoretical study clearly demonstrates that the flexoelectric effect itself becomes suppressed by increasing dielectric anisotropy and rotational viscosity of liquid crystals and this is subject to the fixed flexoelectric coefficients. Therefore, where the improvement on image-quality in practical devices is achieved by the suppression of the flexoelectric effect, the material development should be oriented to that the flexoelectric coefficients are independent on the dielectric anisotropy and the rotational viscosity, that is, the optimization between the dipole moment and anisometric shape of the molecule.

As the time-dependent transmittance curves shown in Fig. 8, the high-dielectric anisotropy and high-rotational viscosity contribute to relatively slow response, but this insensitivity arises with smooth transition between altered frames. This analysis can be supported by comparing the director field with low-dielectric anisotropy and high-dielectric anisotropy in Figs 5(c,d) and 7(b,c), respectively. One can find out the director fields between the frames become similar, especially in the region above the electrode centers. The reason of the smooth transition would be, (1) molecular response, upon broken head-tail symmetry, cannot follow the direction of the field change owing to the high-rotational viscosity; (2) the high-dielectric anisotropy of the liquid crystal suppresses the flexoelectric effect so that the local transmittance on the center of each electrode becomes similar as well as the director field in both frames is not significantly different as shown in Fig. 7(b,c).

Understanding on arising of the twist distortion in the complicated coupling-mechanism with respect to the association upon the direction of the electric field vector and flexoelectric polarization will be appreciated for unveiling of the flexoelectric effect and corresponding optical effect under the lateral electric field application. As long as the shape of electric field is symmetric between the positive and negative frames, theoretically no image-flickering occurs. Therefore, the IPS mode can be the genuine technology for the low-frequency driving liquid crystal display devices aiming the low-power consumption.

## Methods

### Cell

The interdigitated indium-tin-oxide electrodes of width *w* = 5 μm and space *l* = 5 μm on a glass substrate are prepared by photo-lithography; nematic liquid crystals (MLC-6252, Merck) are sandwiched by another glass substrate maintaining the cell gap *d* by 5 μm diameter-sized ball spacers after planar alignment layers are spin-coated on both substrates and rubbed along the direction 7° to the long-direction of the electrodes.

### Electro-optic measurement

The voltage-dependent transmittance is measured with respect to various frequencies (1k, 60, 30, 10, 5, and 1 Hz). The prepared cell is placed between crossed polarizers at the minimum dark level detected by a 1° narrow-angle luminance probe (J6523, Tektronix) with a tungsten-halogen light source (Radiometric fiber optic illuminator 77501, Oriel; filtered by monochromatic wave *λ* = 554 nm) while the alternating current fields are laterally applied in the cell. The measurement is done by an automatized system for voltage-application and photo-detection.

### Polarizing optical microscopy (POM)

POM images in Fig. 3 are taken where the cell is sandwiched by two-crossed polarizers, and the crossed polarizers are rotated while the cell is fixed, securely.

### High-speed camera and POM observation

The POM images are taken using a high-speed camera (Phantom v211, Vision Research) by 200 frames per second (fps) with the exposure time <5 ms, so that series of the frames under positive and negative polarities of the applied electric fields are systematically acquired. By image-processing, the series of the local transmittance in the spatial region of interest, which is indicated by the black arrow in Fig. 1(c), can be plotted.

### Numerical simulation of liquid crystal director and transmittance

The free energy density of the system is taken into account for theoretical study, which consists of elastic free energy density,


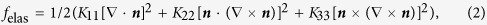


quadratic dielectric coupling,


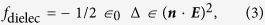


and linear flexoelectric coupling,


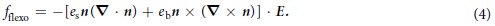


The electric potential distribution is analyzed based on Laplace’s equation and the optical transmittance and gray images are calculated based on the [2 × 2] extended Jones matrix method by a multi-dimensional finite element method (FEM) simulator (TechWiz LCD, Sanayi system).

## Additional Information

**How to cite this article**: Kim, M. S. *et al*. Flexoelectric effect in an in-plane switching (IPS) liquid crystal cell for low-power consumption display devices. *Sci. Rep.* **6**, 35254; doi: 10.1038/srep35254 (2016).

## Supplementary Material

Supplementary Information

Supplementary Video S1

Supplementary Video S2

Supplementary Video S3

Supplementary Video S4
