# Supplementary material for: Flexoelectric effect in an in-plane switching (IPS) liquid crystal cell for low-power consumption display devices
Source: Sci Rep. 2016 Oct 12;6:35254. doi: 10.1038/srep35254 (PMC5059722; doi:10.1038/srep35254)
Supplement: Supplementary Information [file srep35254-s1.pdf]

Supplementary Information

Flexoelectric effect in an in-plane switching (IPS) liquid crystal cell for low-power consumption display devices

Min Su Kim[1,2], Philip J. Bos[1], Dong-Woo Kim[1], Deng-Ke Yang[1], Joong Hee Lee[2], and Seung Hee Lee[2,*]

[1]Liquid Crystal Institute, Kent State University, Kent, OH 44242, United States
[2]Applied Materials Institute for BIN Convergence, Department of BIN Convergence Technology, and Department of Polymer Nano Science and Technology, Chonbuk National University, Jeonju, Jeonbuk 561-756, Korea

[*]lsh1@jbnu.ac.kr

Supplementary Figures

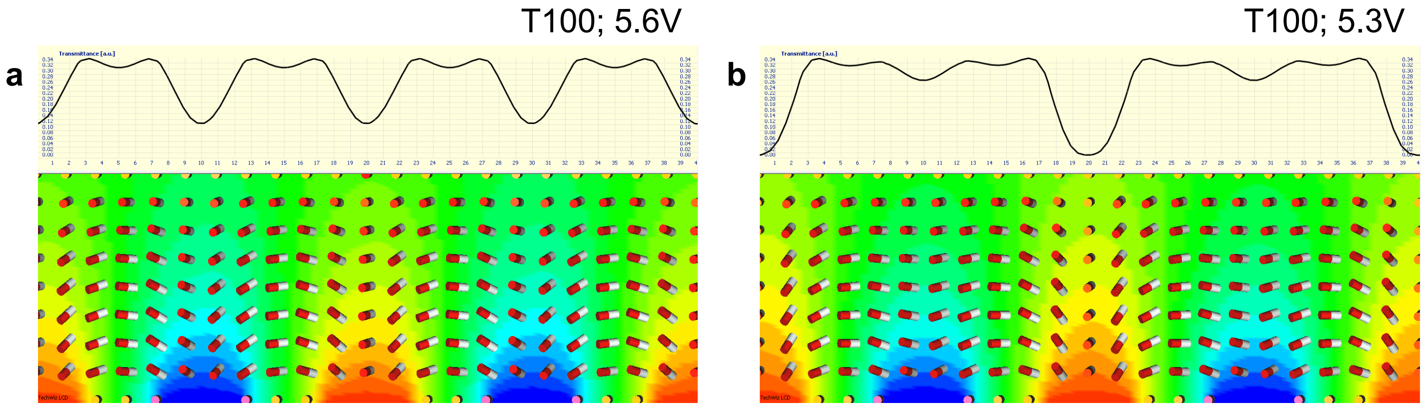

Supplementary Figure S1. Numerical simulation of local transmittance and director fields (a) without (b) with flexoelectric effect (at positive frame) in in-plane switching (IPS) liquid crystal cell at maximum transmittance.

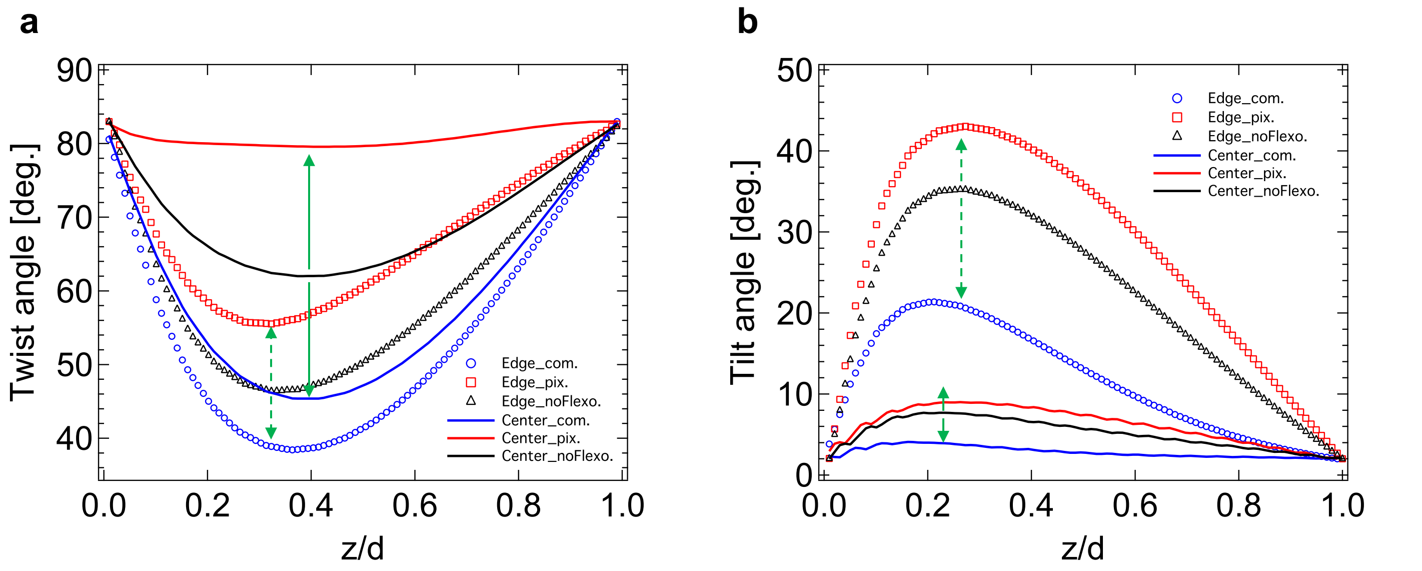

Supplementary Figure S2. Numerical simulation of (a) twist and (b) tilt angle of directors with respect to vertical location in the cell at positive frame. Solid lines are at the centre of the electrode and markers are at the edge of the electrode. At positive frame, the highest plateau of local transmittance curve is shifted to the area above the common electrode, so that local transmittance drops at the area above the pixel electrode as shown in Supplementary Figure S1(b).

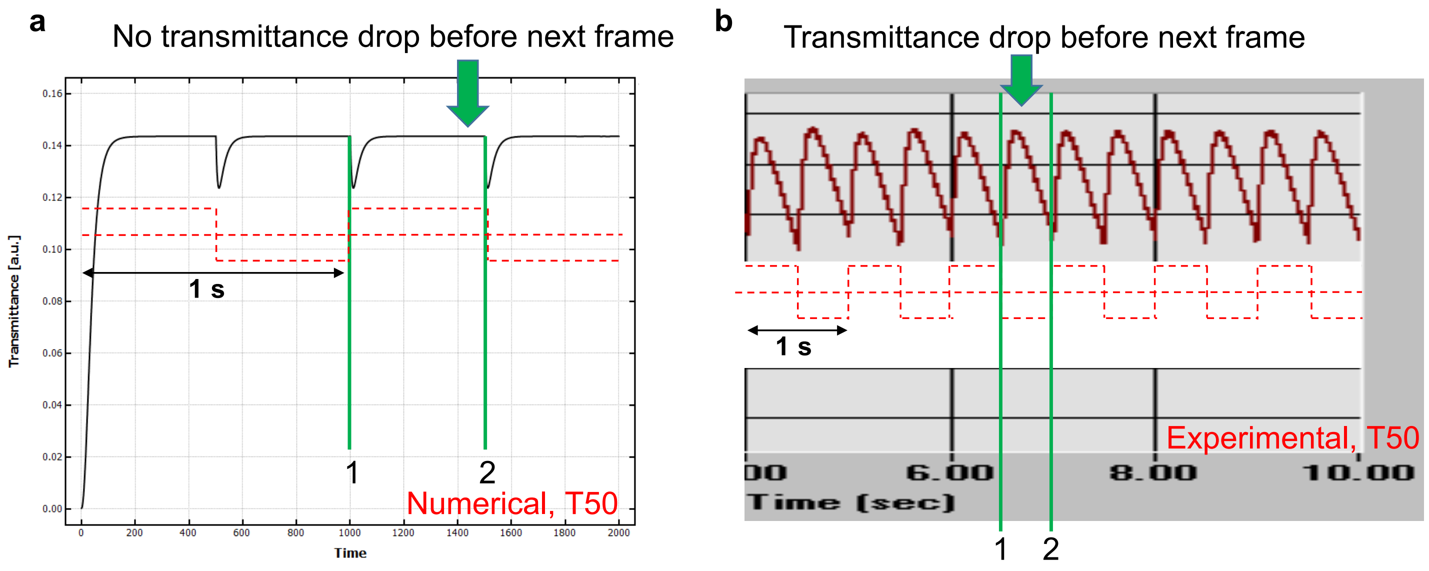

Supplementary Figure S3. Ion (charge impurity) effect shown by comparison between (a) a numerical simulation and (b) an experiment of time-dependent transmittance curves. The electric potential drops due to the ions adsorption and desorption on the electrodes in real cell.

Supplementary Video captions

Supplementary Video S1. Applied electric field at 60 Hz; the frames taken at the frame rate 200 fps; the video playing frame rate of 10fps.

Supplementary Video S2. Applied electric field at 10 Hz; the frames taken at the frame rate 200 fps; the video playing frame rate of 10fps.

Supplementary Video S3. Time evolution of director field in numerical simulation with e_s (e_b) = 0 (0) pC/m.

Supplementary Video S4. Time evolution of director field in numerical simulation with e_s (e_b) = 15 (-5) pC/m.
